# Frequency of putative periodontal pathogens among type 1 diabetes mellitus: a case–control study

**DOI:** 10.1186/s13104-019-4364-3

**Published:** 2019-06-10

**Authors:** Krishnan Mahalakshmi, Ponnudurai Arangannal, Santoshkumari

**Affiliations:** 1 Department of Microbiology/Research Lab for Oral-Systemic Health, Sree Balaji Dental College and Hospital, Bharath Institute of Higher Education and Research, Velachery - Tambaram Road, Chennai, Tamilnadu 600100 India; 2 Department of Paedodontics and Preventive Dentistry, Sree Balaji Dental College and Hospital, Bharath Institute of Higher Education and Research, Velachery - Tambaram Road, Chennai, 600100 India; 3 Department of Paedodontics and Preventive Dentistry, Sri Venkateswara Dental College and Hospital, Thalambur, Chennai, India

**Keywords:** *Campylobacter rectus*, *Eikenella corrodens*, PCR, *Prevotella intermedia*, *Prevotella nigrescens*, Type 1 diabetes mellitus

## Abstract

**Objective:**

The aim of the present study is to compare and assess the risk of periodontitis due to the presence of four putative periodontopathic bacteria viz., *Eikenella corrodens*, *Campylobacter rectus*, *Prevotella intermedia* and *Prevotella nigrescens*. To fulfil the above objective, polymerase Chain reaction using the primers targeting *16S rRNA* gene of the bacterial species was performed with the subgingival plaque collected from the permanent first molars of type 1 diabetic children and age matched healthy children.

**Results:**

The prevalence of periodontal pathogens in diabetic and healthy children was 6% and 16% for *E. corrodens*, 18% and 36% for *C. rectus*, 2% and 2% for *P. intermedia*, 4% and 0%, for *P. nigrescens* respectively. Statistically, significant difference was not observed for the prevalence of all the four periodontal pathogens between type 1 diabetic and healthy children (P = 1.00). The results of the present study thus reveal a negative correlation of type I diabetes to periodontitis in association to *Eikenella corrodens*, *Campylobacter rectus*, *Prevotella intermedia* and *Prevotella nigrescens*.

## Introduction

Type 1 diabetes mellitus (T1DM) is usually diagnosed in children and young adults and was previously known as juvenile diabetes. The diagnosis of type 1 diabetes can be made at any age, but it usually manifests in childhood, adolescence or early adulthood, before the age of 20 [1]. American Diabetes Association has reported type 1 diabetes in 5–10% of patients with diabetes [1]. Periodontal diseases are the sixth most common complication among the varied systemic complications of uncontrolled diabetes [2]. Periodontal disease is caused by Gram-negative anaerobic subgingival microflora [3, 4]. These gram-negative periodontal pathogens have been detected in high frequency in adults with long-duration insulin-dependent diabetics [5]. The prevalence of gram-negative periodontal bacterial pathogens and its positive correlation to periodontal disease have been widely reported in type 2 diabetic population. In addition the subgingival microbiota in diabetes patients with periodontitis generally is similar to that observed in patients with periodontitis who are not diabetic [6–11].

Barring our earlier study, hardly there are any previous reports correlating T1DM and periodontitis with regard to the prevalence of periodontal bacterial pathogens [12]. The aim of the present study was to compare the prevalence and assess the risk of four other key periodontopathic bacteria viz., *Eikenella corrodens*, *Campylobacter rectus*, *Prevotella intermedia* and *Prevotella nigrescens* among the type 1 diabetic children and healthy children/adolescents between the age group of 7 and 14 years, using polymerase chain reaction. identification of these periodontopathic bacteria in children with type 1 diabetes can act as a biomarker for risk of periodontitis.

## Main text

### Methods

The present study was cleared by the institutional ethics committee of Sree Balaji Dental College and Hospital, Chennai and the Department of Endocrinology and Diabetology, Institute of Child Health and Hospital for Children (ICHS), Chennai. Type 1 diabetic children/adolescents (n = 50) were recruited based on the following inclusion and exclusion criteria. Healthy children/adolescents (n = 50) attending the Department of Paedodontics and Preventive Dentistry, Sree Balaji Dental College and Hospital, Chennai were included as control group.

Children with confirmed diagnosis of T1DM, age between 7 and 14 years, the presence of all permanent first molars, were the inclusion criteria. Children with diabetic related systemic complications, children who had professional dental cleaning and periodontal therapy within the last 6 months, antibiotic therapy within the last 3 months and history of orthodontic treatment were excluded. Children with no systemic diseases, age between 7 and 14 years and with the presence of all permanent first molars were the inclusion criteria for the control group. Informed consent was obtained from the parents/guardian of all the children enrolled in the study. Plaque index [13] and gingival index [14] were recorded to assess for periodontitis. Bleeding on probing was more in T1DM children/adolescents. The clinical parameters were recorded by a single examiner (pedodontist) with 5 years of experience in the field. The probing depths ranged between 3 and 5 mm. After removal of supragingival plaque with sterile cotton roll, the subgingival plaque was collected using sterile Gracey curette from the buccal surface of all first permanent molars. The subgingival plaque was pooled in sterile eppendorf tubes containing 500 μl of phosphate buffered saline (pH 7.8). The samples were stored at − 20° until assayed. (Deep Freezer, Blue star).

#### PCR analysis

PCR analysis was carried out in the Microbiology laboratory of Sree Balaji Dental College and Hospital, DNA extraction was performed by boiling-lysis method and the supernatant was used as the template for PCR assay. Species—specific primers for amplification of the target organisms have been used as suggested by previous studies (Table 1) [15]. PCR cocktail (50 μL) for the detection of 16S *rRNA* of the four bacterial species contained 5 μl of 10× PCR buffer (pH 8.4), 1 U Taq DNA polymerase (Bangalore genei, India.), 0.25 mMol/L of each deoxyribonucleotides (dNTP) (Medox Biotech India Pvt., Ltd.), 1.5 mM MgCl2 (Sigma-Aldrich Pvt., Ltd.), 0.5 μM of each primer (Sigma-Aldrich Pvt., Ltd.) and 5 μL of the template. The DNA of bacterial sequences submitted to Gen Bank under accession no’s HQ269826, HQ269827, HQ202263 and HQ202264 were used as positive controls. PCR was carried out for 35 cycles with each cycle consisting of denaturation (94 °C for 5 min), annealing (50 °C for 1 min) and primer extension (72 °C for 1.5 min). Amplified PCR products were analyzed in 1.5% agar gel electrophoresis in Tris–Borate-EDTA buffer. The gel was stained with 0.5 µg/mL ethidium bromide and photographed under BioRad UV gel documentation system. 100 bp ladder (Medox Biotech India Pvt Ltd) served as the molecular weight marker.

**Table 1:** Species specific primers for PCR assay [15]

Primer pairs (5′–3′), *16S rRNA*	Amplicon length in bp
*Eikenella corrodens*	
CTAATACCGCATACGTCCTAAG	688
CTACTAAGCAATCAAGTTGCCC	
*Campylobacter rectus*	
TTTCGGAGCGTAAAACTCCTTTTC	598
TTTCTGCAAGCAGACACTTTT	
*Prevotella intermedia*	
TTTGTTGGGGATAAAGCGGG	575
TCAACATCTCTGTATCCTGCGT	
*Prevotella nigrescens*	
ATGAAACAAAGGTTTCCGGTAAG	804
CCCACGTCTCTGTGGGCTGCGA	

The P-value was obtained by Fisher’s exact test (two-tailed). SPSS software 15 was used for all statistical analysis. In the present study, P < 0.05 was considered statistically significant. The odds ratio at 95% confidence interval was calculated to assess the association of the bacterial species to type I diabetes.

### Results

The subgingival plaque samples of type I diabetic children and healthy children amplified the *16S rRNA* gene of *E. corrodens*, *C. rectus*, *P. intermedia* and *P. nigrescens* in the product size of 688 bp, 598 bp, 575 bp and 804 bp respectively. None of the samples showed non-specific amplification. Table 2 shows the clinical parameters measured. A significant difference was not noticed among the T1DM and control group with regard to PD. The Plaque index was 0.35 and 0.37 for T1DM and healthy group respectively. While the gingival index was 0.88 and 0.44 for T1DM and healthy group respectively. The level of gingival bleeding (gingival index—0.88) on probing was slightly high in T1DM children. The HbA1c level of children with T1DM ranged between 5.66 and 11.50%. About 45% of the T1DM children HbA1c readings were above 8.0%. The duration of diabetes among T1DM subjects ranged 3–7 years. Table 3 shows the prevalence of four bacterial species in type I diabetic group and healthy subjects. Three of type I diabetic children showed co-occurrence of *E. corrodens* and *C. rectus*. While in healthy children five samples showed co-occurrence of *E. corrodens* and *C. rectus*. All four organisms were not present in the same subject’s subgingival plaque. T The association between T1DM and healthy subjects is not statistically significant with respect to prevalence of all the four bacterial species screened.

**Table 2:** Measured clinical parameters in the study group and healthy subjects

Clinical parameters	T1DM/healthy range	T1DM/healthy mean	P value
Plaque index (PI)	0.2–0.6/0.2–0.6	0.3/0.3	1.000
Gingival index (GI)	0.6–0.9/0.2–0.7	0.8/0.4	1.000
HbA1c %	5.66–11.50/NA	8.9/NA	NA
Duration of diabetes	3–7 years/NA	5 years/NA	NA

**Table 3:** Prevalence of four periodontopathic bacterial species among the study group and healthy subjects

Bacterial species	Type 1 diabetic group (n = 50) (%)	Healthy group (n = 50) (%)	P value
*Eikenella corrodens*	3 (6)	8 (16)	0.199
*Campylobacter rectus*	9 (18)	18 (36)	0.071
*Prevotella intermedia*	1 (2)	1 (2)	1.000
*Prevotella nigrescens*	2 (4)	0 (0)	0.495

The odds ratio (at 95% confidence interval)/risk ratio calculated for *E. corrodens*, *C. rectus*, *P. intermedia* and *P. nigrescens* were 0.3351/0.375, 0.3902/0.5, 1/1 and ∞/∞ respectively.

### Discussion

Earlier studies have documented that diabetes may interfere with progression of periodontal disease [16–18]. The point that periodontal disease may complicate the severity of diabetes by worsening the degree of glycemic control has also been established [19, 20]. It is a well-recognised fact that diabetes mellitus is a risk factor for progression of periodontitis in adults [2]. Among the long term complication periodontitis is a 6th most complication for uncontrolled diabetes. Gram negative anaerobes are implicated in the etiopathogenesis of periodontitis and hence identification of putative periodontal pathogens can act as a marker for onset of periodontitis. Although culture techniques are gold standard, cultivating these obligate anaerobes are very difficult as they lose viability once exposed to oxygen, which may result in error report. Hence, PCR technique was chosen to screen for these periodontal pathogens.

The association between periodontal disease and diabetes mellitus with respect to gram-negative anaerobes in the adult population has been widely studied [13, 21, 22]. While data regarding the prevalence of periodontopathic bacteria among type I diabetic children are lacking. A very low prevalence of *P. intermedia* was observed in T1DM and healthy cohorts. This is in line with Mohamed et al. [13] study but for type 2 diabetic groups. Similarly *P. nigrescens*, a black pigmented putative periodontal pathogen was present only in two samples of the T1DM group. While *P. nigrescens* was completely absent among healthy children. No correlation was observed with the presence of four putative periodontopathic bacteria viz., *E. corrodens*, *C. rectus*, *P. intermedia* and *P. nigrescens* to bleeding on probing and probing depth among type 1 diabetes. Although significant difference was not observed between the type I diabetes and healthy controls with regard to the prevalence of the bacterial species screened, there was increased bleeding on probing in the gingiva of type 1 diabetic children. This finding was similar to two previous reports on periodontal changes among type I diabetic children [23, 24].

The odds ratio clearly reveals a negative correlation to the prevalence of key periodontal bacterial pathogens and T1DM. However, the present study reports a very low prevalence of these anaerobic bacteria in children/adolescents. This finding shows that Children with T1DM are not at risk of developing periodontitis due to these anaerobic bacterial species as against the adults with type 2 diabetes The concurrent finding among type I diabetic and healthy children in the present study with respect to bacterial profiles is well in agreement with Lalla et al. [7]. The prevalence of *P. intermedia* in the present study does not corroborate with the study of Okada et al. [25] who has reported significantly higher prevalence among healthy children. An absence of significant statistical difference between type 1 diabetic and healthy cohort with regard to the prevalence of *E. corrodens*, *C. rectus*, *P. intermedia* and *P. nigrescens* is well in agreement with various other previous reports [5, 8, 10, 25–27]. The present paper emphasizes a negative association of the screened periodontal pathogens to periodontitis among T1DM children. Several earlier observational studies have reported the risk of periodontitis among type 2 diabetes mellitus on the basis of clinical parameters. While, the present study highlights a negative correlation to T1DM with regard to the presence of putative periodontopathic bacterial species in the subgingival plaque of children. Further studies with large sample size are needed to correlate an association between T1DM and periodontitis. To conclude, the results of the present study suggest that type 1 diabetes has no significant influence on the prevalence periodontal bacterial species screened. The results show an absence of risk to periodontitis due to these pathogens in T1DM children.

## Limitations

- Large follow-up studies to screen for array of periodontal pathogens to study their association to type 1 diabetes among children and adolescents.
- *16S rRNA* metagenomic analysis of subgingival plaque may further help to trace the potential effect of type 1 diabetes on bacterial composition of dental plaque.

## Data Availability

In view of participants’ confidentiality, the raw data would not be shared. The research data are available in the main document.

## References

[1] Silverstein J, Klingensmith G, Copeland K, Plotnick L, Kaufman F, Laffel L (2005). Care of children and adolescents with type 1 diabetes: a statement of the American Diabetes Association. Diabetes Care.

[2] Löe H (1993). Periodontal disease. The sixth complication of diabetes mellitus. Diabetes Care.

[3] Socransky SS, Haffajee AD, Cugini MA, Smith C, Kent RL (1998). Microbial complexes in subgingival plaque. J Clin Periodontol.

[4] Socransky SS, Haffajee AD (2000). Periodontal microbial ecology. Periodontol.

[5] Thorstensson H, Dahlén G, Hugoson A (1995). Some suspected periodontopathogens and serum antibody response in adult long-duration insulin-dependent diabetics. J Clin Periodontol.

[6] Ebersole JL, Holt SC, Hansard R, Novak MJ (2008). Microbiologic and immunologic characteristics of periodontal disease in Hispanic Americans with type 2 diabetes. J Periodontol.

[7] Lalla E, Kaplan S, Chang SM, Roth GA, Celenti R, Hinckley K (2006). Periodontal infection profiles in type 1 diabetes. J Clin Periodontol.

[8] Campus G, Salem A, Uzzau S, Baldoni E, Tonolo G (2005). Diabetes and periodontal disease: a case–control study. J Periodontol.

[9] Hintao J, Teanpaisan R, Chongsuvivatwong V, Ratarasan C, Dahlen G (2007). The microbiological profiles of saliva, supragingival and subgingival plaque and dental caries in adults with and without type 2 diabetes mellitus. Oral Microbiol Immunol.

[10] Tervonen T, Oliver RC, Wolff LF, Bereuter J, Anderson L, Aeppli DM (1994). Prevalence of periodontal pathogens with varying metabolic control of diabetes mellitus. J Clin Periodontol.

[11] Davila-Perez C, Amano A, Alpuche-Solis AG, Patiño-Marin N, Pontigo-Loyola AP, Hamada S (2007). Distribution of genotypes of *Porphyromonas gingivalis* in type 2 diabetic patients with periodontitis in Mexico. J Clin Periodontol.

[12] Arangannal P, SanthoshKumari, Krishnan P, Nichani MH, Krishnan M, Chamarthi V (2013). Detection of putative periodontopathic bacteria in type 1 diabetic and healthy children: a comparative study. Indian J Dent Res.

[13] Silness J, Loe H (1964). Periodontal disease in pregnancy. II Correlation between oral hygiene and periodontal condition. Acta Odontol Scand.

[14] Löe H (1967). The Gingival Index, the Plaque Index and the Retention Index Systems. J Periodontol..

[15] Ashimoto A, Chen C, Bakker I, Slots J (1996). Polymerase chain reaction detection of 8 putative periodontal pathogens in subgingival plaque of gingivitis and advanced periodontitis lesions. Oral Microbiol Immunol.

[16] Cianciola LJ, Park BH, Bruck E, Mosovich L, Genco RJ (1982). Prevalence of periodontal disease in insulin dependent diabetes mellitus (juvenile diabetes). J Am Dent Assoc.

[17] Nelson RG, Shlossman M, Budding LM, Pettitt DJ, Saad MF, Genco RJ (1990). Periodontal disease and NIDDM in Pima Indians. Diabetes Care.

[18] Thorstensson H, Hugoson A (1993). Periodontal disease experience in adult long duration insulin dependent diabetics. J Clin Periodontol.

[19] Grossi SG, Genco RJ (1998). Periodontal disease and diabetes mellitus; two way relationship. Ann Periodontol.

[20] Donnahue R, Wu T (2001). Insulin resistance and periodontal disease: an epidemiologic overview of research needs and future directions. Ann Periodontol.

[21] American Diabetes Association (2012). Diagnosis and classification of diabetes mellitus. Diabetes Care.

[22] Silvestre FJ, Miralles L, Llambes F, Bautista D, Solá-Izquierdo E, Hernández-Mijares A (2009). Type I diabetes mellitus and periodontal disease: relationship to different clinical variables. Med Oral Patol Oral Cir Bucal.

[23] Lalla E, Cheng B, Lal S, Tucker S, Greenberg E, Goland R (2006). Periodontal changes in children and adolescents with diabetes: a case–control study. Diabetes Care.

[24] Pinson M, Hoffman WH, Garnick JJ, Litaker MS (1995). Periodontal disease and type I diabetes mellitus in children and adolescents. J Clin Periodontol.

[25] Okada M, Hayashi F, Nagasaka N (2001). PCR detection of 5 putative periodontal pathogens in dental plaque samples from children 2 to 12 years of age. J Clin Periodontol.

[26] Mohamed Hasaan Gassim, Idris Shaza Bushra, Mustafa Manal, Ahmed Mutaz Faisal, Åstrøm Anne Nordrehaug, Mustafa Kamal, Ibrahim Salah Osman (2016). Influence of Type 2 Diabetes on Prevalence of Key Periodontal Pathogens, Salivary Matrix Metalloproteinases, and Bone Remodeling Markers in Sudanese Adults with and without Chronic Periodontitis. International Journal of Dentistry.

[27] Sastrowijoto SH, Hillemans P, van Steenbergen TJ, Abraham-Inpijn L, Graaff J (1989). Periodontal condition and microbiology of healthy and diseased periodontal pockets in type 1 diabetes mellitus patients. J Clin Periodontol.

